# Summary of the National Advisory Committee on Immunization (NACI) Statement—Recommendations on Fractional Influenza Vaccine Dosing in the Event of a Shortage: Pandemic preparedness

**DOI:** 10.14745/ccdr.v49i04a01

**Published:** 2023-04-01

**Authors:** Angela Sinilaite, Pamela Doyon-Plourde, Kelsey Young, Robyn Harrison

**Affiliations:** 1 NACI Secretariat, Public Health Agency of Canada; 2 NACI Influenza Working Group Chair at the time of the NACI Statement writing; 3 University of Alberta, Alberta Health Services, Edmonton, AB

**Keywords:** National Advisory Committee on Immunization, NACI, vaccination, influenza vaccine, intradermal, fractional dose

## Abstract

**Background:**

At the commencement of a pandemic, it is important to consider the impact of respiratory infections on the health system and the possibility of vaccine shortages due to increased demand. In the event of an influenza vaccine shortage, a strategy for administration of fractional influenza vaccine doses might be considered. This article reviews the available evidence for efficacy, effectiveness, immunogenicity and safety of fractional influenza vaccine dosing, and summarizes the National Advisory Committee on Immunization (NACI) recommendations on fractional dosing strategies by public health programs in Canada.

**Methods:**

Two rapid literature reviews were undertaken to evaluate the efficacy, effectiveness, immunogenicity and safety of fractional influenza vaccine dosing via the intramuscular or intradermal route. The NACI evidence-based process was used to assess the quality of eligible studies, summarize and analyze the findings, and apply an ethics, equity, feasibility and acceptability lens to develop recommendations.

**Results:**

There was limited evidence for the effectiveness of fractional influenza vaccine dosing. Fractional dosing studies were primarily conducted in healthy individuals, mainly young children and infants, with no underlying chronic conditions. There was fair evidence for immunogenicity and safety. Feasibility issues were identified with intradermal use in particular.

**Conclusion:**

NACI recommended that, in the event of a significant population-level shortage of influenza vaccine, a full-dose influenza vaccine should continue to be used, and existing vaccine supply should be prioritized for those considered to be at high risk or capable of transmitting to those at high risk of influenza-related complications or hospitalizations. NACI recommended against the use of fractional doses of influenza vaccine in any population.

## Introduction

Influenza vaccination in Canada is provided annually through provincial and territorial seasonal influenza vaccine programs. Although provincial and territorial influenza vaccine programs vary across the country, all programs cover individuals who are at high risk of severe outcomes due to influenza and individuals that are capable of transmitting influenza to those at high risk (e.g. household members, healthcare workers). Due to the rapid timelines required for vaccine production each year, any significant impact to the manufacturing process may cause delays in influenza vaccine delivery or decrease the overall number of doses produced, potentially resulting in vaccine shortages for a season. A significant and unexpected increase in demand for the influenza vaccine could also lead to insufficient supply, as the number of doses available is based on orders made primarily in the spring months in advance of the next influenza season. This could be particularly relevant at pandemic times, as it was for the 2020–2021 influenza season when increased demand for seasonal influenza vaccine was observed in the southern hemisphere as a result of the coronavirus disease 2019 (COVID-19) pandemic. A strategy for the administration of fractional influenza vaccine doses (i.e. less than a full-dose) might be considered in these situations, as the use of fractional doses would provide vaccine programs the ability to vaccinate a larger number of people with the amount of vaccine that is available when supply is limited. The objective of this advisory committee supplemental statement is to review the available evidence for efficacy, effectiveness, immunogenicity, and safety of fractional influenza vaccine dosing, and to provide guidance on potential fractional dosing strategies in the event of a significant influenza vaccine shortage in Canada.

In Canada, influenza vaccines are currently authorized for intramuscular (IM) administration only, apart from the live-attenuated influenza vaccine (LAIV), which is administered intranasally. Intradermal (ID) administration is not covered within influenza vaccine product monographs and would therefore be considered off-label. For the purposes of these recommendations, the National Advisory Committee on Immunization (NACI) considered two different fractional dosing strategies: 1) fractional IM administration of influenza vaccine; and 2) fractional ID administration of influenza vaccine.

## Methods

To inform NACI’s recommendations, two rapid literature reviews were undertaken by the Methods and Applications Group for Indirect Comparisons (MAGIC) through the Drug Safety and Effectiveness Network (DSEN) on the topic of fractional influenza vaccine dosing. The rapid review methods were specified *a priori* in a written protocol that included the research questions, search strategy, inclusion and exclusion criteria, and quality assessment. The NACI Influenza Working Group reviewed and approved the protocol. The search strategies were developed in consultation with an experienced librarian based on pre-defined population, intervention, control, outcomes, study design and timeframe, and the following research questions ((1,2)): What is the safety and effectiveness of using fractional dosing strategies to deliver IM seasonal influenza vaccines?; and What is the safety and effectiveness of using fractional dosing strategies to deliver seasonal influenza vaccine by ID administration?

The reviews were completed by MAGIC, with additional data extraction (notably immunogenicity outcomes as indirect evidence for effectiveness for IM administration of fractional doses) completed by the Public Health Agency of Canada (PHAC). For both reviews, EMBASE and MEDLINE electronic databases, Cochrane Library, Cochrane Central Register of Controlled Trials and international clinical trial registries were searched for IM vaccine publications in the last 20 years and ID vaccine publications in the last 10 years. Searches were restricted to articles published in English. Additionally, hand-searching of the reference lists of included articles and relevant systematic reviews was performed.

For the ID fractional dose review, the DSEN MAGIC team conducted all data extraction and performed a meta-analysis for effectiveness, immunogenicity, and safety outcomes. The risk of bias for the included ID studies was assessed using the Cochrane risk-of-bias tool for randomized trails.

For the IM fractional dose review, the DSEN MAGIC team extracted and narratively summarized the data for effectiveness and safety, and provided PHAC with a list of studies that assessed immunogenicity outcomes to be used as indirect evidence for effectiveness for IM administration of fractional doses. PHAC technical staff then extracted the immunogenicity data from these studies and summarized the evidence narratively. The level of evidence (i.e. study design and methodological quality of studies) included in the IM review were assessed independently by two reviewers with PHAC using the design-specific criteria outlined by Harris *et al.* ((3)).

A systematic assessment of ethics, equity, feasibility, and acceptability of influenza vaccine fractional dosing strategies was also conducted according to established NACI methods ((4)).

The body of evidence of benefits and harms was synthesized and analyzed according to NACI evidence-based process ((5)) to develop recommendations. Following thorough review of the evidence, NACI formulated, reviewed and approved recommendations. Full details and results are presented in the NACI *Recommendations on Fractional Influenza Vaccine Dosing* ((6)).

## Results

Key characteristics of the studies included in the DSEN MAGIC team reviews and additional analyses by PHAC are summarized in **Table 1**.

**Table 1:** Characteristics of included studies providing evidence related to the comparative efficacy, effectiveness, and immunogenicity of fractional vs. full-dose influenza vaccine for intramuscular and intradermal administration

Author, year	Study design (vaccine)	Study population and setting	Outcomes
**Intramuscular**			
Antony *et al.*, 2020	Scoping review including RCTs, non-RCTs and observational studies Standard dose inactivated influenza vaccine	Individuals of all ages 10 RCTs presented data relevant for the systematic review (3 in adults and 9 in children)	Local, systemic and/or severe AEs
Robertson *et al.*, 2019	RCT 2016–2017 influenza season (7.5 mcg vs. 15 mcg dose of IIV4)	Healthy children 6–35 months of age • 7.5 mcg group (n=682) • 15 mcg group (n=682) US multi-centre study	Difference in seroconversion rate (15 mcg group–7.5 mcg group) post-vaccination GMT ratios (15 mcg group–7.5 mcg group) post-vaccination Local, systemic and/or severe AEs
Jain *et al.*, 2017	RCT 2014–2015 influenza season (7.5 mcg vs. 15 mcg dose of IIV4-Fluzone quadrivalent)	Healthy children 6–35 months of age • 7.5 mcg group (n=1,028) • 15 mcg group (n=1,013) Multi-centre study conducted in the US and Mexico	Seroprotection 28 days (or 56 days for unprimed individuals) post-vaccination Seroconversion 28 days (or 56 days for unprimed individuals) post-vaccination GMT rise 28 days (or 56 days for unprimed individuals) post-vaccination Local, systemic and/or severe AEs
Halasa *et al.*, 2015	RCT October 5, 2010 and March 2, 2012; the studies were conducted before the 2010–2011 and 2011–2012 influenza seasons (7.5 mcg vs. 15 mcg dose of IIV3-Fluzone)	Healthy children 6–35 months of age Primed individuals: 7.5 mcg group (n=9) and 15 mcg group (n=21) Naïve individuals: 7.5 mcg group (n=55) and 15 mcg group (n=119) US multi-centre study	Seroprotection 28 days (naïve individuals 28 days after 2^nd^ dose of influenza vaccine) post-vaccination Seroconversion 28 days (naïve individuals 28 days after 2^nd^ dose of influenza vaccine) post-vaccination Difference in GMT (15 mcg group–7.5 mcg group) 28 days after last vaccination Local, systemic and/or severe AEs
Pavia-Ruz *et al.*, 2013	RCT 2008–2009 influenza season (7.5 mcg vs. 15 mcg dose of IIV3-Fluarix or Fluzone)	Healthy children 6–35 months of age • Fluarix 7.5 mcg group (n=1,017) • Fluarix 15 mcg group (n=1,013) • Fluzone 7.5 mcg group (n=1,031) Multi-centre study conducted in the US, Hong Kong, Mexico, Thailand and Taiwan	Seroprotection 28 days (or 56 days for unprimed children) post-vaccination Seroconversion 28 days (or 56 days for unprimed children) post-vaccination GMT rise post-vaccination Local, systemic and/or severe AEs
Langley *et al.*, 2012	RCT 2008–2009 influenza season (7.5 mcg vs. 15 mcg dose of IIV3-Flulaval or Vaxigrip)	Healthy children 6–35 months of age • Flulaval 7.5 mcg group (n=164) • Flulaval 15 mcg group (n=167) • Vaxigrip 7.5 mcg group (n=43) Canadian multi-centre study	Seroprotection 28 days post-vaccination Seroconversion 28 days post-vaccination GMT ratios (Flulaval 15 mcg/Flulaval 7.5 mcg) 28 days post-vaccination (adjusted for prior influenza vaccination, baseline titre—pooled variance) Local, systemic and/or severe AEs
Della Cioppa *et al.,* 2011	RCT 2008–2009 influenza season (7.5 mcg vs. 15 mcg dose of IIV3 or IIV4)	Healthy children 6–35 months of age • IIV3 vaccine recipients: 7.5 mcg group (n=25), 15 mcg group (n=22) and IIV3-Vaxigrip 15 mcg group (n=26) • IIV4 vaccine recipients: 7.5 mcg group (n=25) and 15 mcg group (n=28) Multi-centre study conducted in Finland and Belgium Note: only a subset of study groups relevant for this review are presented in the systematic review	Seroprotection on day 50 Seroconversion on day 50 GMT rise on day 50 Local, systemic and/or severe AEs
Skowronski *et al.*, 2011	RCT 2008–2009 influenza season (7.5 mcg vs. 15 mcg dose of IIV3-Vaxigrip)	Healthy children 6–23 months of age • 7.5 mcg group (n=124) • 15 mcg group (n=128) Canadian multi-centre study	Seroprotection 27–45 days after the 2^nd^ dose Seroconversion 27–45 days after the 2^nd^ dose GMT rise after the 2^nd^ dose Local, systemic and/or severe AEs
Chi *et al.*, 2010	RCT 2007–2008 influenza season (9 mcg vs. 15 mcg dose of IIV3-Fluzone)	Adults 65 years of age and older without serious or unstable conditions • 9 mcg group (n=64) • 15 mcg group (n=65) US study	Seroprotection four weeks post-vaccination Local, systemic and/or severe AEs
Engler *et al.*, 2008	RCT 2004–2005 influenza season (7.5 mcg vs. 15 mcg dose of IIV3-Fluzone)	Healthy adults 18–64 years of age • 7.5 mcg group: 18–49 years old (n=284) and 50–64 years old (n=276) • 15 mcg group: 18–49 years old (n=274) and 50–64 years old (n=280) US multi-centre study	RR of one or more medical visits for ILI involving the upper or lower respiratory tract Difference in seroconversion 21 days post-vaccination Difference in seroprotection 21 days post-vaccination Local, systemic and/or severe AEs
Belshe *et al.*, 2007	RCT 2006–2007 influenza season (3 mcg, 6 mcg, 9 mcg and 15 mcg doses of IIV3-Fluzone)	Healthy adults 18–49 years of age • 3 mcg group (n=29) • 6 mcg group (n=30) • 9 mcg group (n=32) • 15 mcg group (n=31) US single-site study	Seroconversion rate 28 days post-vaccination Seroprotection rate 28 days post-vaccination Local, systemic and/or severe AEs
Kramer *et al.*, 2006	RCT 2004–2005 influenza season (7.5 mcg vs. 15 mcg dose of IIV3-Fluzone)	Healthy adults healthcare workers 18 years of age and older • 7.5 mcg group (n=222) • 15 mcg group (n=222) US single-site study	RR of clinical diagnosis of influenza (ILI) for individuals vaccinated with a 7.5 mcg dose compared to 15 mcg vaccine dose Proportion of clinical diagnosis that was laboratory-confirmed influenza infection
**Intradermal**			
Egunsola *et al.*, 2020	Rapid review and meta-analysis including RCTs, non-RCTs and observational studies (ID administration of a 9 mcg vs. IM dose of 15 mcg of HA per influenza vaccine strain)	Individuals of all ages • 13,759 participants from RCTs • 164,021 participants from observational studies	RR of influenza infection and/or ILI from the ID administration of a 9 mcg of HA per strain dose of influenza vaccine compared to 15 mcg of HA per strain IM dose RR of seroconversion rate of ID compared to standard dose of IM administration RR of seroprotection rates for ID compared to standard dose of IM administration Risk of local AEs with ID compared to IM administration Risk of systemic AEs following vaccination with ID compared to IM vaccine

Abbreviations: AE, adverse event; GMT, geometric mean titre; HA, hemagglutinin; ID, intradermal; IIV3, trivalent inactivated influenza vaccine; IIV4, quadrivalent inactivated influenza vaccine; ILI, influenza-like-illness; IM, intramuscular; mcg, microgram; RCT, randomized controlled trial; RR, risk ratio; US, United States

## Vaccine efficacy and effectiveness

### Fractional intramuscular dosing (efficacy/effectiveness)

Two randomized clinical trials (RCTs) ((7,8)) were identified that assessed the efficacy of fractional IM administration of a 7.5 mcg of hemagglutinin (HA) per strain dose versus a 15 mcg of HA per strain dose of the trivalent inactivated influenza vaccine (IIV3) against influenza-like-illness (ILI) during the 2004–2005 influenza season in the United States (US). Both studies were deemed to be good quality according to the criteria outlined by Harris *et al.* ((3)). The studies did not demonstrate a difference in efficacy between the full-dose (15 mcg) and the half-dose (7.5 mcg) of IIV3 against ILI.

### Fractional intradermal dosing (efficacy/effectiveness)

Two studies ((9,10)) assessed the efficacy of fractional ID administration of influenza vaccine against laboratory-confirmed influenza infection or ILI in adults using IIV3. A meta-analysis of these two RCTs studies found no significant difference in the risk of influenza infection/ILI from the ID administration of a 9 mcg of HA per strain dose of influenza vaccine compared to 15 mcg of HA per strain IM dose (pooled risk ratio [RR]: 0.61, 95% CI, 0.19–1.91).

## Immunogenicity

Overall, 10 RCTs and one meta-analysis of 16 RCTs reported immunogenicity outcomes for fractional doses of IM or ID influenza vaccine administration. The immunogenicity outcomes assessed by these studies included geometric mean-fold rise in hemagglutination inhibition (HI) titres (i.e. ratio of post to pre-vaccination geometric mean titre), seroprotection rate (i.e. proportion of participants with HI titres of at least 40 post-vaccination) and seroconversion rate (i.e. proportion of participants with at least a four-fold increase in HI titres post-vaccination, HI titre increase from less than 10 pre-vaccination to at least 40 post-vaccination, or both).

### Fractional intramuscular dosing (immunogenicity)

Ten articles ((8,11–19)) were identified that assessed immunogenicity outcomes for fractional doses of influenza vaccines administered IM. All ten studies were RCTs deemed to be of good quality according to the Harris *et al.* criteria ((3)). Of these studies, two ((8,11)) were conducted in adults within the age range of 18 and 64 years and one ((13)) was conducted in adults of 65 years of age and older. The other seven studies ((13–19)) were all conducted in children within the age range of 6 to 35 months.

One study ((8)) in adults reported that the study groups that received a fractional dose of 7.5 mcg of HA per strain had statistically lower proportions of seroconversion and seroprotection post-vaccination than those who received the full-dose. Four studies ((15–17,19)) that statistically assessed the difference in immunogenicity between a full-dose and a half dose of influenza vaccine in children 6 to 35 months of age reported mixed results. Additional studies (one in adults and two in children) ((13,17,19)) that assessed varying fractional doses of influenza vaccine (3 mcg, 6 mcg, 7.5 mcg and 9 mcg of HA per strain) found that as the dose of influenza vaccine decreased, the immunogenic response also decreased. However, lower doses continued to meet criteria set for non-inferiority despite the reduced response compared to full-dose (according to current US Food and Drug Administration or previous European Medicines Agency criteria).

### Fractional intradermal dosing (immunogenicity)

A meta-analysis ((2)) included 16 RCTs studies that assessed immunogenicity outcomes for fractional doses of influenza vaccine administered ID. The meta-analysis demonstrated no significant difference in the seroconversion rates for the study groups that had received fractionated doses (3 mcg, 6 mcg, 7.5 mcg or 9 mcg of HA per strain) by ID administration compared to 15 mcg of HA per strain dose given IM for all influenza strains. A meta-analysis was also performed for seroprotection rates compared to a full-dose of 15 mcg of HA strain per IM dose and found no significant difference for groups that received ID administration at doses of 3 mcg, 7.5 mcg or 9 mcg of HA per strain. Similarly, there was no significant difference in seroconversion or seroprotection rates between older adults that had received the fractional 9 mcg of HA per strain ID dose compared to those that received the full 15 mcg of HA per strain IM dose. However, seroprotection rates were significantly lower for those that had received a dose of 6 mcg of HA per strain for influenza A(H1N1) compared to a full IM dose.

## Safety

### Safety of the intramuscular route of administration

The rapid review identified seven studies ((13–19)) that assessed safety outcomes (local, systemic and severe adverse events [AEs]) of fractional IM influenza vaccine in infants or toddlers in the range of 6 to 36 months of age. Three studies were identified in the rapid review that assessed safety of fractional IM influenza vaccination in adults: two of the studies ((8,11)) involved adults between the ages of 18–64 years (18–49 years and 18–65 years) and one study ((12)) included adults older than 65 years of age.

### Safety of intradermal route of administration

Twenty-three studies ((9,10,12,20–39)) were identified that assessed the safety of ID administration of influenza vaccine and were able to be included in a meta-analysis performed by the DSEN MAGIC team. The studies identified included various fractional doses (3 mcg, 6 mcg, 9 mcg of HA per strain), as well as a full non-fractional dose (i.e. 15 mcg of HA per strain) of ID-administered influenza vaccine. Overall, there was fair evidence that fractional doses of influenza vaccine administered via the IM and ID routes do not result in a significant difference with regard to severe systemic AEs post-influenza vaccination. No significant increases in pain have been reported with ID influenza vaccine administration compared to IM administration; however, the risk of local AEs, such as ecchymosis, erythema, pruritus and swelling occurring post-vaccination at the injection site, is significantly higher with ID administration of influenza vaccine compared to IM administration.

## Feasibility

Several feasibility issues were identified when considering fractional dosing of current influenza immunizations or administration of ID doses of influenza vaccines. Administering a fractional IM or ID dose would require administering a lower volume of vaccine to achieve the desired lower dose, which is only possible when influenza immunizations have been packaged as multi-dose vials and not as pre-filled syringes. The ID administration of vaccine requires a different gauge needle than IM administration, multi-dose vials (which are not always available midway in the season if supplies run low), and training and skill in ID administration that not all vaccinators will have. Significant training would also be required to ensure vaccinators are equipped in advance to provide ID influenza vaccinations and feel comfortable doing so. The number of vaccinators who are authorized and able to provide ID vaccination also vary by jurisdiction.

The volume of vaccine to be administered is high even if using a fractional dose and would therefore require two ID injections if regular needles and syringes were used rather than just one. The majority of studies of administration of influenza vaccine by the ID route used micro-needle injectors for administration, which are not yet authorized or widely available in Canadian settings. Furthermore, the use of fractional doses is not covered within influenza vaccine product monographs and would therefore require a novel communication and consent plan for any off-label dosing if it were adopted. Finally, implementation of such an ID immunization program would require structured monitoring for any potential modification to a seasonal influenza vaccine program running low on vaccine and advanced planning would have to factor this in *a priori* as multi-dose vials are not always available midway in the season.

National Advisory Committee on Immunization recommendations for public health program decision-making

1. NACI recommends that, in the event of a significant population-level shortage of influenza vaccine, a full-dose influenza vaccine should continue to be used, and existing vaccine supply should be prioritized for those considered to be at high risk or capable of transmitting to those at high risk of influenza-related complications or hospitalizations. ***(Strong NACI Recommendation)***

• NACI concluded that there is fair evidence to recommend the use of a full-dose influenza vaccine (15 mcg or 60 mcg HA per strain, dependent on vaccine product) compared to a fractional dose for individuals at high risk or those capable of transmitting to those at high risk of influenza-related complications or hospitalizations. ***(Grade B Evidence)***

2. NACI recommends against the use of fractional doses of influenza vaccine in any population. ***(Discretionary NACI Recommendation)***

• NACI concluded that there is insufficient overall evidence at this time to recommend the use of fractional IM influenza vaccine doses. ***(Grade I Evidence)***

• NACI concluded that there is fair evidence that fractional ID influenza vaccine doses provide a sufficient immune response, but this route of administration is not feasible at this time. ***(Grade B Evidence)***

The detailed findings of the two rapid literature reviews, rationale and relevant considerations for these recommendations can be found in the NACI Statement, *Recommendations on Fractional Influenza Vaccine Dosing* ((6)).

## Conclusion

In the event of a significant population-level shortage of the currently available influenza vaccine products, NACI recommends that full-dose influenza vaccine should continue to be used and existing vaccine supply should be prioritized for those considered to be at high risk or capable of transmitting to those at high risk of influenza-related complications or hospitalizations. NACI recommends against the use of fractional doses of influenza vaccines in any population.
